# Navigating fierce love during the pandemic: Reflections of a Pinay Scholar Warrior

**DOI:** 10.1177/1473325020984151

**Published:** 2021-03

**Authors:** Alma M Ouanesisouk Trinidad

**Affiliations:** School of Social Work, Portland State University, Portland, USA

**Keywords:** Social work teaching, service, pandemic, women of color in the academy

## Abstract

This article presents reflections of a Pinay Scholar Warrior of Kapu Aloha and Mahalaya during the pandemic. Excerpts from her social media and reflections from her personal journal reveal the complexities of navigating fierce love in social work teaching and service. Issues related to facilitating critical analyses of social determinants of health, grief and loss, and rediscovering strengths and joys. Implications of women of color in the academy is alluded.

The global COVID-19 pandemic of 2020 created strong tidal waves and shocks to many people in the world, especially the most vulnerable among us in communities of color. Being the first and only Filipina-American tenured professor at a predominantly white institution in the Pacific Northwest, I take a stance as a Pinay Scholar Warrior of Kapu Aloha and Mahalaya. Pinay denotes being a Filipina-American. Scholar Warrior is the use of my position as a scholar through its three pillars of scholarship/research, teaching, and service to fight for, protect, and push for the advancement of ideals and values of *kapu aloha (sacred love* in Hawaiian*)* and *mahalaya (freedom* and *love* in Tagalog). In essence, it is putting forth the praxis of radical and fierce love in action through the use of one’s power and privilege of being a scholar. This stance has urged me to refine and redefine, continuously, this practice as ongoing threats heighten to the people I serve during the pandemic.

For the purpose of this article, I share my critical reflections generated from excerpts from my social media and personal journal entries. I focus on the complexities of navigating the struggles and pain, aiming to uphold fierce love to serve the people, and how it takes a high level of discipline and clarity. I name this process of navigation as a “Warrior Dance.” When one dances, one bends back and forth, sometimes dancing alone or dancing with partners or with a collective. It is this dance, I hope to realize my sense of agency, voice, and compassion for self, so I can sustain my participation and engagement in multiple social movements in the long haul.

## The current social positionalities of the scholar warrior

The pandemic reveals my importance and sacredness of my current leadership roles in the academy. I am reminded of how being a first generation professor carries weight and emotional burden, often keeping in check my sense of responsibility and accountability to the most vulnerable among the community. This requires a dance, a warrior dance of calling-out and calling-in culture. It is this dance that keeps me humbly grounded.

Calling-out “describes the act of publicly naming instances of oppressive language and behavior” and calling-in “can be a powerful tool to [authentically together] address those mistakes and create space for real change and positive impact” (Mahan, 2017). There is a time and place to call-out people and institutions. As a scholar activist, I do this a lot! Through advocacy, I point out the communities’ needs, the disparities that exist, and the attempt to change the paradigm by exerting the community voices and rights. The warrior harnesses the fighting energy for the rights of the people. Simultaneously, in my role as *part of* the system, institution, and/or organization that serves or holds gatekeeping powers, I am often called-in to be part of the change from within, in the peripheral, or hybridity. Since the pandemics of COVID-19 and heightened awareness of police brutalities on Black people, this dance of doing both in different spaces has become more profound to me. I reflect upon this warrior dance, in hopes we can better provide opportunities to refine a discipline of the praxis of radical love.

## At the dawn of the COVID-19 pandemic: The drum beat begins the warrior dance

The week our country officially declared COVID-19 as a global pandemic, I taught my last sessions of winter quarter:

March 12, 2020: This past Tuesday night was my last session of the term for BSW Advocacy for Policy Change … We had a “gratitude” circle. I love how my class has become a deep learning community for each other, expressing high level of intellectual curiosity, care as struggles were faced, and collaboration. They were inspired by the diverse speakers I pulled in who are part of social movements and change. I am hopeful for the future. Big thanks to TP from the Malaya Movement PNW & General Consul JE from the Federated States of Micronesia Consulate for sharing your life work! Topic of the week on March 4: Internationalization and Globalization.

I always called-in people from the ground to be guest speakers in my courses. They are people I’ve built relationships with, and are part of social movements in the Asian Pacific Islander communities. More recently, I became involved with the Malaya Movement, a Filipino-American movement aimed to stop the killings and dictatorship, and stand for democracy in the Philippines. My role as a professor is to use my teaching, research, and service as a platform to integrate Filipino issues more explicitly and call-out growing injustices. Juxtapost, I became interested in the role of embassies and consulates in playing a role in upholding human rights or calling-out injustices imposed by U.S. imperialism. The examination of local and global relationships helped “beat the drums” quite differently.

The weeks throughout March, I began to mourn the loss of routines. It encouraged me to deepen my analysis of the global pandemic and its impact on communities of color, particularly immigrants and refugees, and posts included:

March 14: will never know how it feels to experience martial law or house arrest. It sure feels like it on a more “luxurious” fashion.

To multitask–grading, virtual work meetings, responding to students, etc., tend to some “volunteer” related tasks, and “homeschool” (or manage or set parameters and new routines for the kids)–is challenging.

Needing to remind my elder parents in Hawai'i to practice physical social distancing (e.g., don't go to a funeral; don't go to Costco if not needed), as they are in the high risk group, especially my mom. Doing this from afar is challenging.

This is our new reality. Worrying for the most vulnerable among us. Trying to stay calm. May we strive as a community and society.

[I posted this artwork the same day.]

March 26: A pretty spot on analysis. We are observing the ugliness of neoliberal capitalism. We need to rise as humanity. Pulling the power/mana of the people ground up and our ancestral spirits …
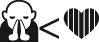

“This is a crisis which is not being experienced evenly. Inequality, and oppression, are magnified as the rich can take precautions which are unavailable to the poor” (Darroch, 2020).

March 28: Heartbreaking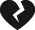. Our health system is broken. People over profit. Strained existing “public” resources. Denied care. Neglect. Systemic neglect & care.

We can do better! We must rise from this.
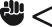

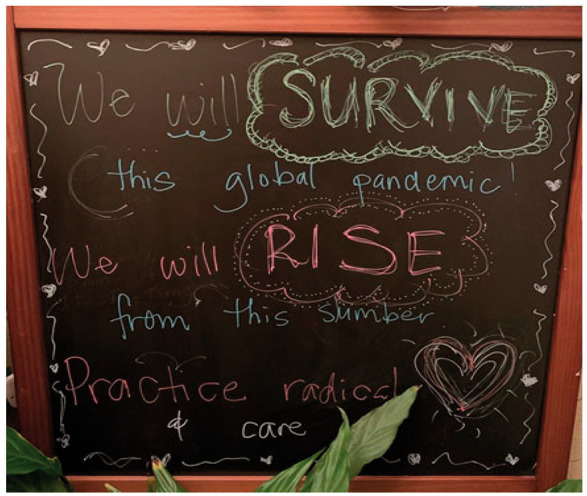

My doodle on March 30, 2020.

## Drum beating loader in the warrior dance: The pandemic moves

During the month of April, we witnessed drastic changes in policies and procedures. Remote learning for both of my kids was imposed. Both of us parents worked full time from home, which blurred the boundary lines. How were we going to do this? Extended family members in Hawai‘i are essential workers. The constant worrying, coupled with caring for my aging parents from afar, became intense. I constantly wondered how to manage the disruptions:

April 18: The professoriate career path has always been unequal and this pandemic makes it clearer of such challenges. Hence, why promotion and tenure processes must change. The same for any career advancement and performance review process in any career that WOC embark in.

“This pandemic can teach some of us an important lesson: mothers and fathers together are facing a short-term reorganization of care and work time. In the long run, these changes in productivity will affect careers. Those with fewer care duties are aiming for the stars. Will anyone in the academic community take into account our unbalanced approach to family care and work? No. All of us will participate together in open competition for promotion and positions, parents and non-parents alike.

Academic work … is basically incompatible with tending to children” (Minello, 2020).

As a professor/scholar activist, I’m taking the time to document my joys during the pandemic, as it keeps me grounded:

*witnessing such keen and sharp analysis from students on the critique of capitalism and its harm on our communities and questioning our roles as social workers

*deep discussions during zoom student mtgs on such topic areas —yoga+homesteading = reclaiming ways of life & coping with trauma; diversity, inclusion, & equity issues on board membership & how it plays out in the non profit work in the current conditions of the pandemic

*research myntoring mtgs integrate the following topic areas = Filipino migrant issues & decolonizing mental health in context of capitalism through family histories; unpacking family memories & trauma through photos; capturing career pathways of migrating and settling in the US.

Yes! These discussions with my students and myntees bring me joy of critical consciousness. It’s moving for all of us.

April continued to present more mourning–loss of a colleague, death anniversaries of loved ones; increased COVID-19 rates among Pacific Islanders, including Filipino communities locally and nationally. Simultaneously, it was a month to call-in our ancestral wisdom and resilience.

April 25: Pausing as it's been a great couple days, even if I'm grieving, slightly unmotivated, and continuously disgusted by the interlocking of the systems of oppression and people who hold great power over our communities.

.Truly amazed and heartfelt by the coming of community as we mourn the loss of R.O., a dear friend from my PhD years.

…How a critical mass of my students are experiencing a lot, and to hold space/place for that is both delicate, hard, and draining. Collective trauma is intense. I so appreciate how the human component can help us rise to continuous learning, and making the present the experience.

…How I am still so extremely active in my advocacy and leadership in multiple spaces! There are a lot of local and global issues that touch the community, and recognizing that small and big changes are ever moving. I'm particularly happy that May is recognized for API month in North Clackamas County School District. My entire family put forth a testimony in a short amount of time, and had it be read during a board meeting is something!

## The drum beats harder: The warrior dance becomes fiercer

May and June came in full force as we celebrated the end of the academic year. The pandemic was getting worse, and more explicit calls were rising for racial equity in organizations I serve as a board member. Statements to call-out police brutalities, coupled with the pandemic, required a keen look at the organization's role in alleviating harm. Simultaneously, as a professor, I was called-out for creating “rupture” and dismissal of the feelings of pain and struggles in the classroom. The dance got more intense, and required such discipline to be brave and humbled. The stamina to be vulnerable, open, and human was tested:

June 3: Today was especially heavy. Trauma is deep. Facilitating learning in higher ed has been exhausting, especially during the COVID-19 pandemic and the intensity of violence among our black and Indigenous communities. I know the ancestors are strongly present and with me to guide me as I do this work, because I deeply FEEL and ever so sharply keep analyzing with humility.

It pains me to bear witness to my students’ experiences. It pains me when I am “called-out.” I also know it comes from a place of deep sorrow, rage, struggle, and anger. I also know that I cannot be the only one that facilitates healing. I am not sure I trust the current systems to elevate healing among our peoples. How do we step up our sense of responsibility?. Please keep me and us who serve in deep prayers. This anguish is unsettling and necessary.

June 12: Things I had with me. Groundings. I marched today to Disarm PSU Now. When Black Lives Matter interweaves with the global pandemic of COVID 19. Channeling the energy of the resistance of Indigenous & Lumad communities in the Philippines, my motherland, on Philippines Independence Day. As a Pinay/Filipina, born and raised on Molokai, settled in white Oregon on stolen lands and displaced Indigenous communities, and holding all of the positionalities I have, still questioning concepts of independence, democracy, freedom, and livelihood. The self interest of the privileged in the realm of capitalism is the greatest toxicity that suffocates us all. Need to breathe in and out new air. We are molding the healing future of 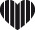.

Symbols with ironies. My 10 years of service at PSU pinned on bleeding black heart (to remember Jason Washington; the man killed by PSU officer); Filipino flag linked to “Cultural Resource Centers” (which if an institution authentically honored BIPOC, such centers would not be needed); Lumad necklace with yellow hearts with my PSU keys and ID (which at one point did not let me go back into the building I work in). I also lost my “kapu aloha” button gifted to me sometime between the pandemic. I hope whoever found it will embrace its energy. Sometimes contradictions reveal things, and help us imagine new futures.

## The warrior dance’s purpose of sustainability and perseverance

The invisible labor that comes from being a woman of color scholar is exhausting, as it intertwines to my livelihood. Always in contradiction. Even being tenured, the power struggles are burdensome. The bend of calling-out and calling-in people and processes comes with new discoveries of joy, realizing collective energies, and putting forth our boundaries. The warrior dance must be revisited, revised, redefined to be more inclusive of myntoring our *own* first and foremost, and bridging solidarities across.


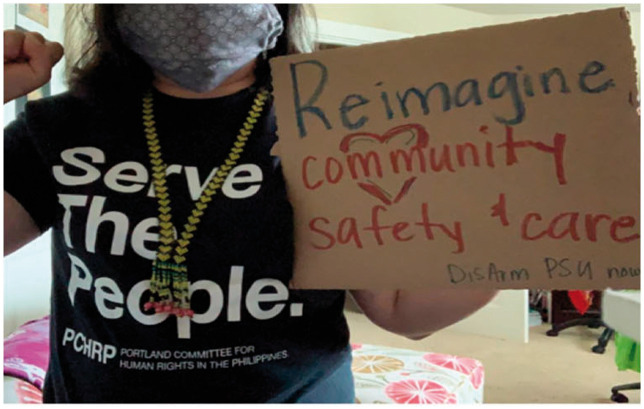
 
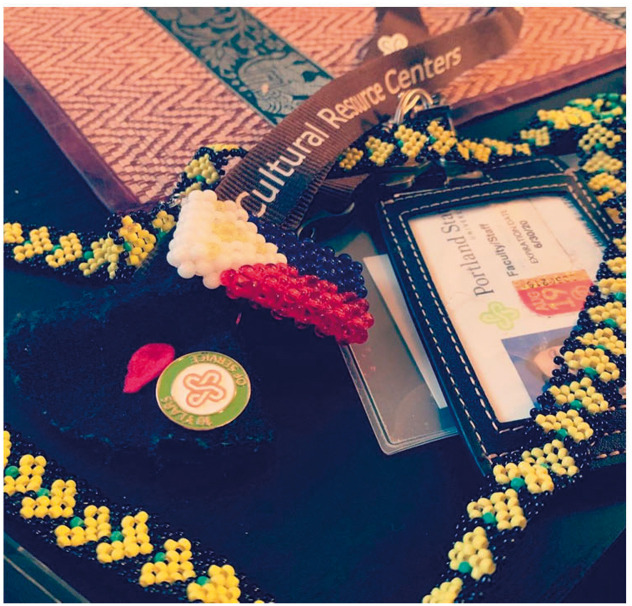


The last four months reveal volcanic-like eruptions of the pandemic. As I posted on July 1, this is my hope and dream for the future of our social work education, teaching, and research:

The praxis of RADICAL love involves being able to struggle out the conflicts, tensions, pain, and contradictions. We are not taught to be DISCIPLINED in such. We need opportunities to learn how to do this. So hard to do when individualism, perfectionism, trauma, and exploitation (all related to dehumanizing) run deep. This needs to change. Overthrow the system and culture that feed individualism, perfectionism, trauma, and exploitation.

May we learn the warrior dance together, being able to bend to and fro with grace and humility!
